# Bioinformatics Analysis to Screen Key Targets of Curcumin against Colorectal Cancer and the Correlation with Tumor-Infiltrating Immune Cells

**DOI:** 10.1155/2021/9132608

**Published:** 2021-11-12

**Authors:** Xinyue Han, Chao Yang, Cui Guo, Yimin Xu, Xiaoqiang Liu, Runnan Xie, Xiangxue Meng, Zhihong Cheng, Xiaoling Fu

**Affiliations:** ^1^Second Department of Oncology, Yueyang Hospital of Integrated Traditional Chinese and Western Medicine, Shanghai University of Traditional Chinese Medicine, Shanghai 200437, China; ^2^Liaoning University of Traditional Chinese Medicine, Shenyang 100021, Liaoning, China; ^3^Department of Pain, Shibei Hospital, Shanghai 200435, China; ^4^China State Institute of Pharmaceutical Industry, National Pharmaceutical Engineering Research Center, Shanghai 201203, China

## Abstract

**Purpose:**

Curcumin is a potential drug for the treatment of colorectal cancer (CRC). Its mechanism of action has not been elucidated. This study aims to investigate the mechanism of action of curcumin in the treatment of CRC via bioinformatics methods such as network pharmacology and molecular docking.

**Methods:**

The targets of curcumin and CRC were obtained from the public databases. The component-targets network of curcumin in the treatment of CRC was constructed by Cytoscape v3.7.2. Through protein-protein interaction (PPI), the Gene Ontology (GO), and the Kyoto Encyclopedia of Genes and Genomes (KEGG), important targets and signaling pathways related to CRC treatment were identified. Finally, the results were verified by molecular docking, and the correlation between the key targets and tumor-infiltrating immune cells (TICs) was analyzed.

**Results:**

A total of 30 potential targets of curcumin for CRC treatment were collected. The GO function enrichment analysis showed 140 items, and the KEGG pathway enrichment analysis showed 61 signaling pathways related to the regulation of protein kinase activity, negative regulation of apoptosis process, cancer signaling pathway, and PI3K-Akt signaling pathway. The molecular docking results showed that curcumin could be combined with AKT1, EGFR, and STAT3 more stably, and AKT1 has the strongest binding to curcumin. Bioinformatics analysis discovered that the expression of core targets AKT1, EGFR, and STAT3 in CRC was related to TICs.

**Conclusion:**

This study explored the targets and pathways of curcumin in the treatment of CRC. The core targets are AKT1, EGFR, and STAT3. The study indicated that curcumin has preventive and treatment effects on CRC through multitarget and multipathway, which laid the foundation for follow-up research.

## 1. Introduction

Colorectal cancer (CRC) is a complex heterogeneous disease involving multiple genes and epigenetic factors [1]. According to [2], 147950 new CRC cases resulted in 53200 deaths in the United States. The incidence of CRC is the top three in all tumors, and mortality is the second, which seriously endangers human health [3, 4]. At present, surgery, chemotherapy, radiotherapy, targeted therapy, and immunotherapy are the mainstream treatments for CRC. However, long-term use of these therapies leads to serious side effects, including nausea and vomiting, oral ulcer, diarrhea, bone marrow suppression, and immunosuppression. Drug resistance commonly occurs in CRC [5, 6]. For patients with advanced CRC, there is no effective treatment [7]. The side effects and drug resistance of chemotherapy adversely affect the quality of life, treatment process, treatment results, and treatment costs of patients [8]. Therefore, exploring new drugs and targets for the treatment of CRC has attracted more and more attention.

**Figure 1:**
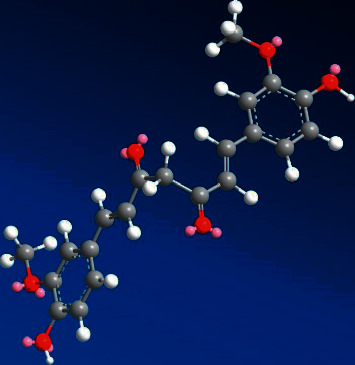
The 3D structure of curcumin.

With the development of traditional Chinese medicine, its monomeric active ingredients have become a focus of recent research. For example, curcumin, matrine, paclitaxel, and so on have been reported for the prevention and treatment of CRC. In recent years, more and more studies have shown that curcumin has great potential in the treatment of CRC [9–13]. *Curcuma longa* L., commonly known as turmeric, is a rhizomatous herb of the ginger (Zingiberaceae) family. It can promote blood and qi circulation and relieve pain. Curcumin is a lipophilic polyphenol compound extracted from the Zingiberaceae family, which lowers blood glucose and has anticancer, anti-inflammation, and antiaging effects [14]. The 3D structure of curcumin is shown in Figure 1. Howells et al. confirmed that curcumin is a safe and well-tolerated adjuvant chemotherapy drug for folinic acid/5-fluorouracil/oxaliplatin chemotherapy (FOLFOX) chemotherapy of metastatic CRC [15]. In a clinical trial, curcumin has been proved to promote the transformation of Treg cells to Th1 cells and enhance the production of interferon-*γ*, supporting the antitumor effect of curcumin in CRC [16]. The clinical efficacy of curcumin in the treatment of CRC has been recognized. Subsequent in vivo experiments confirmed that curcumin can reduce inflammation and CRC formation in mouse models [17]. Upregulation of miR-200c and downregulation of EPM5 can inhibit EMT in CRC to prevent or delay the progression of CRC [18]. It can also block G2/M and G1 cycles to inhibit cell growth and induce apoptosis of colorectal cancer cells [19]. These research results showed that curcumin has many potential effects and has definite therapeutic effects on CRC. However, most of the previous studies focused on some signaling pathways and related targets, and they did not comprehensively and systematically explain the mechanism of action of curcumin in the prevention and treatment of CRC that limits the promotion and secondary development of curcumin.

**Figure 2:**
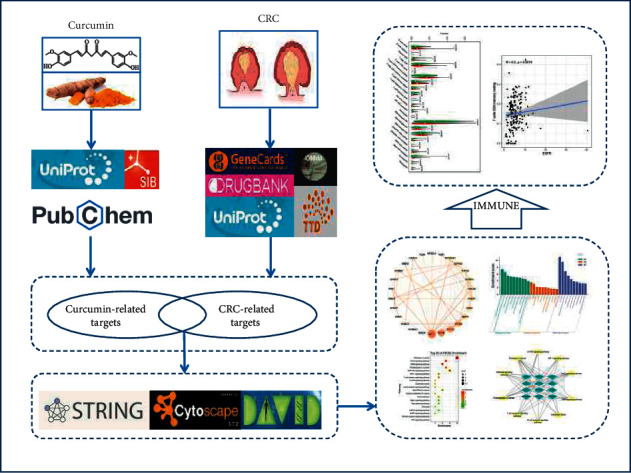
Study framework based on an integration strategy of network pharmacology.

With the development of bioinformatics, network pharmacology can systematically and comprehensively reveal the relationship between the active components of traditional Chinese medicine and its potential mechanism of action. Network pharmacology has become an efficient method for the study of traditional Chinese medicine [20]. This study aims to explore the potential targets and molecular mechanisms of curcumin in the treatment of CRC by network pharmacology and molecular docking analysis. Firstly, we screened the molecular targets of curcumin and pathological targets of CRC by databases. Then, the enrichment analysis was carried out according to the Gene Ontology (GO) and the Kyoto Encyclopedia of Genes and Genomes (KEGG). The multidimensional network of “drug-target-pathway-disease” was constructed by Cytoscape v3.7.2. Finally, the interaction between curcumin and targets was verified by molecular docking analysis, and the correlation between the key targets and tumor-infiltrating immune cells (TICs) was analyzed. The summary of this study is shown in the flowchart of Figure 2.

## 2. Materials and Methods

### 2.1. Data Preparation

#### 2.1.1. Druglikeness Prediction

Lipinski's rule of five (RO5) is an empirical rule for screening potential oral drugs by evaluating the properties of drugs, including molecular weight (MW), octanol-water partition coefficient (XLogP3), polar surface area, number of rotatable bonds, hydrogen bond acceptor count, and hydrogen bond donor count [21]. To explore the druglikeness properties of curcumin, we searched the PubChem database (https://pubchem.ncbi.nlm.nih.gov/) with “curcumin” as the keyword, obtained the SMILES format of curcumin, and then uploaded it to the SwissADME website (https://www.swissadme.ch/) to find relevant parameters.

### 2.2. Collection of CRC-Related Targets

CRC-related targets were downloaded from five public database sources, including GeneCards database (https://www.genecards.org), OMIM database (https://www.omim.org/), TTD database (https://db.idrblab.net/ttd/), UniProt database (https://www.uniprot.org/), and Drugbank database (https://go.drugbank.com/). Species was selected as “*Homo sapiens*” and the keyword as “colorectal cancer.” Then, targets in the pathogenesis of CRC were collected.

### 2.3. Collection of Curcumin-Related Targets

PubChem database was used to obtain the SMILES format of curcumin, which was imported into SwissTargetPrediction database (https://www.swisstargetprediction.ch/). “*Homo sapiens*” was selected, and “probability > 0” was used as the screening condition for target prediction. Finally, the standard gene names were collected by the UniProt platform.

### 2.4. Common Target Screening and Network Construction of Curcumin and CRC

The curcumin-related targets and CRC-related targets were analyzed by Jvenn online platform (https://jvenn.toulouse.inra.fr/app/index.html) to obtain intersection targets and draw the Wayne diagram. The composition-targets network figure was constructed by Cytoscape v3.7.2.

### 2.5. Construction of Protein-Protein Interaction (PPI) Network

The intersection targets were imported into the String platform (https://www.string-db.org/). Then, the interaction relationship between the targets was obtained and saved as the TSV format file. The file was imported into Cytoscape v3.7.2 to get the network diagram. To identify the central nodes and key proteins in the PPI network, the topology parameters were calculated by NetworkAnalyzer, and the degree of centrality (betweenness, closeness, and subgraph) was determined by the CytoNCA.

### 2.6. GO Function and KEGG Pathway Enrichment Analysis

The common targets of curcumin and CRC obtained by the above screening were imported into the DAVID database (https://david.ncifcrf.gov/). The species was set to be “*Homo sapiens.*” With *P* < 0.05 as the statistical difference screening condition, the potential targets of curcumin on CRC were evaluated. The biological function and pathways of the targets were analyzed. Histograms and bubble charts are produced through the Bioinformatics cloud platform (https://www.bioinformatics.com.cn/, an online platform for data analysis and visualization). Then, the targets-pathways network was constructed by Cytoscape v3.7.2.

### 2.7. Molecular Docking Analysis

Molecular docking is a validation method, which simulates the binding of receptors and ligands by computer and predicts their affinity. The mol2 file of curcumin was downloaded from the TCMSP database (https://tcmspw.com/tcmsp.php). The AutoDock Tools 1.5.6 software was imported and saved in *pdbqt* format. The 3D structures of key target proteins were downloaded from the PDB database (https://www.rcsb.org), and the water molecules and inactive ligands were removed by PyMOL software. The protein was imported into AutoDock Tools 1.5.6 software for hydrogenation and charge treatment, and the output was *pdbqt* format. Finally, AutoDock VINA software was used to simulate the molecular docking of the receptor and its ligand, and the optimal binding conformation was obtained. The docking results were visualized by Discovery Studio Visualizer and PyMOL. Molecular docking was used to verify the binding ability of curcumin and the targets, with Matrine as the control. Matrine has a good therapeutic effect on CRC, and its antitumor activity has been verified in a variety of tumors, including CRC [22–24].

### 2.8. Immune Cell Infiltration Analysis

We obtained 437 cases of patients with CRC (39 normal sample cases and 398 tumor samples cases) of the transcriptome RNA-seq data from the TCGA database (https://portal.gdc.cancer.gov/). CIBERSORT algorithm was used to analyze the proportion of TICs, and 22 kinds of TICs maps of CRC samples were constructed. CIBERSORT is a deconvolution algorithm based on normalized gene expression profiles that have been validated by fluorescent-activated cell classification (FACS) and can be used to characterize the composition of 22 TICs in complex samples [25]. Wilcoxon rank-sum test was used to accurately assess the difference in infiltration density among different EGFR, AKT1, and STAT3 levels. Meanwhile, we used the TIMER database (https://cistrome.shi.nyapps.IO/TIMER/) to determine the correlation between screened genes and TICs.

## 3. Results

### 3.1. Druglikeness Analysis of Curcumin

The SMILES format of curcumin (COC1 = C(C = CC( = C1)C = CC( = O)CC( = O)C = CC2 = CC( = C(C = C2)O)OC)O) was imported into swissADME website to obtain relevant parameters. According to Lipinski's rule of five, a drug-like compound should have a molecular weight of less than 500 g/mol, a polar surface area (PSA) of less than or equal to 140 Â, a computed octanol/water partition coefficient (XLogP3-AA) of less than 5, less than 10 rotatable bonds (RB), no more than 10 hydrogen bond acceptor (HBA), and no more than 5 hydrogen bond donors (HBD) [26]. It can be seen from the obtained parameters that the properties of curcumin comply with the RO5, indicating that it has good drug-like properties (Table 1).

### 3.2. Composition-Targets Network

**Figure 3:**
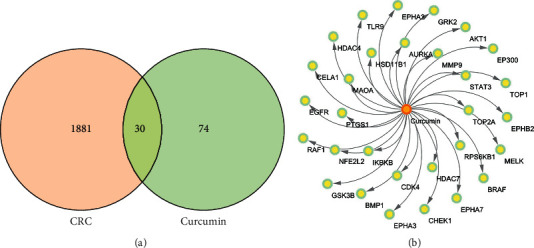
Common targets network. (a) Intersection of curcumin-related targets and CRC-related targets. (b) Composition-targets network.

The related targets of curcumin were searched, 104 targets were obtained after removing the duplication, and 1911 CRC-related targets were obtained after removing the duplication. Next, 30 common targets were screened out, which were considered potential targets of curcumin in the treatment of CRC (Figure 3(a)). The composition-targets network was constructed by Cytoscape v3.7.2 (Figure 3(b)).

### 3.3. Construction of Protein-Protein Interaction Network (PPI)

**Figure 4:**
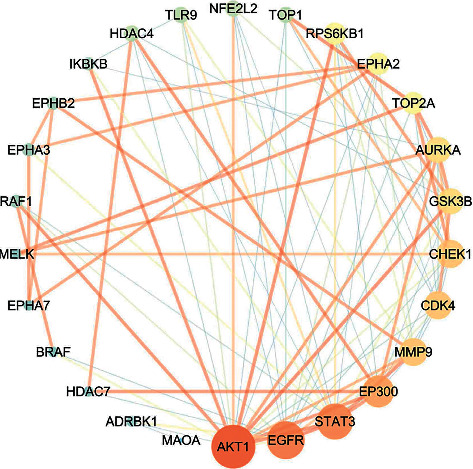
Protein-protein interaction network. The size of the circle represents the node degree of the target protein.

We uploaded 30 common targets to the String database to determine their functional relationships and interactions. Then, the protein interactions with the default confidence level of 0.4 were imported into Cytoscape v3.7.2 to generate a protein-protein interaction (PPI) network, which consisted of 26 nodes and 90 edges, as shown in Figure 4.

To identify the pivot nodes and essential proteins in the PPI network, the topology parameters of the node degree were calculated by the network analyzer, and the three centralities (betweenness, closeness, and subgraph) were determined through the CytoNCA as shown in Table 2.

### 3.4. GO and KEGG Pathway Enrichment Analysis

**Figure 5:**
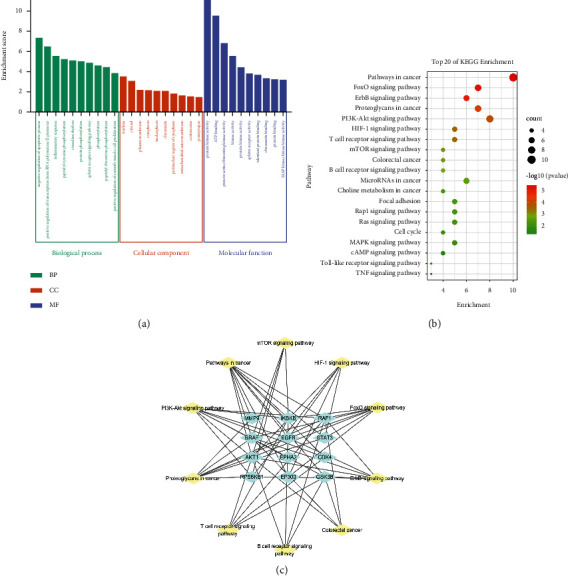
(a) GO enrichment analysis. The top 10 biological processes, the top 10 cellular components, and the top 10 molecular functions. (b) KEGG enrichment analysis; the top 20 KEGG pathways. The color scales indicate the different thresholds for the *p* values, and the sizes of the dots represent the number of genes corresponding to each term. (c) Pathways-targets network.

The GO and KEGG enrichment analysis were performed via DAVID platform. The GO enrichment analysis is composed of biological process (BP), cellular component (CC), and molecular function (MF). A total of 140 items, BP: 93, CC: 14, MF: 33, were obtained by GO functional analysis. The results showed that the effects of curcumin were related to protein kinase activity, ATP binding, negative regulation of apoptotic process, protein serine/threonine kinase activity, and so on, as shown in Figure 5(a).

In the enrichment analysis of the KEGG pathway, 61 enrichment results were obtained. A total of 20 typical pathways were selected to make the visualized bubble diagram after excluding irrelevant pathways (Figure 5(b)). The results showed that these pathways were mainly related to pathways in cancer, PI3K and Akt signaling pathway, FOXO signaling pathway, and so on. Six targets (AKT1, RAF1, BRAF, EGFR, IKBKB, and STAT3) in the first 20 pathways participated in a high frequency (≥9 times), indicating that they played important roles in CRC. Ten representative signaling pathways are selected to construct a “pathways-targets” network, as shown in Figure 5(c).

### 3.5. Molecular Docking

**Figure 6:**
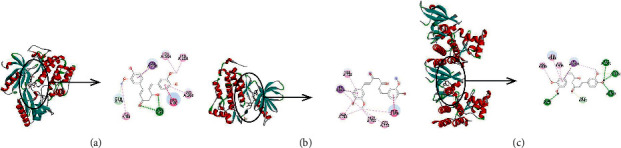
Three-dimensional and two-dimensional representation of the interaction between curcumin and key targets. (a) AKT1; (b) EGFR; (c) STAT3.

Curcumin is docked with three important targets: AKT1, STAT3, and EGFR. These targets are selected not only because they are the key nodes of PPI network but also because they play important roles in KEGG enrichment pathways. The binding energies of AKT1, STAT3, and EGFR with curcumin were −9.9 kcal/mol, −8.7 kcal/mol, and −8.5 kcal/mol, respectively. The binding energies of matrine to AKT1, STAT3, and EGFR were −7.8 kcal/mol, −8.7 kcal/mol, and −7.6 kcal/mol, respectively (Table 3). It can be seen that curcumin has a strong binding force with key targets. The binding of curcumin with AKT1 is mainly through the hydrogen bonding with amino acid residues ASN53 and GLN79, hydrophobic interaction with TRP80, and *π* bonding with LEU210, LEU264, LYS268, VAL270, and ILE84. The binding of curcumin with EGFR is mainly through the hydrophobic interaction with amino acid residues VAL762, PHE856, ALA743, MET790, CYS775, and *π*-bond interaction with LEU844. The binding of curcumin with STAT3 is mainly through the hydrogen bonding of amino acid residues ASP1021, ASN1008, ARG1007, GLU957, GLY962, hydrophobic interaction with LEU881, VAL889, ALA906, and *π* bonding with LEU1010 (Figure 6).

### 3.6. Immune Cell Infiltration Analysis

**Figure 7:**
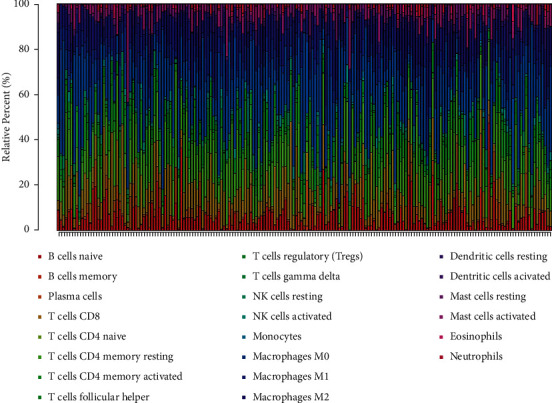
The proportion of 22 TICs in CRC tumor specimens estimated by the CIBERSORT algorithm. Each bar graph shows the cell proportion of each patient, and various colors represent 22 immune cells.

**Figure 8:**
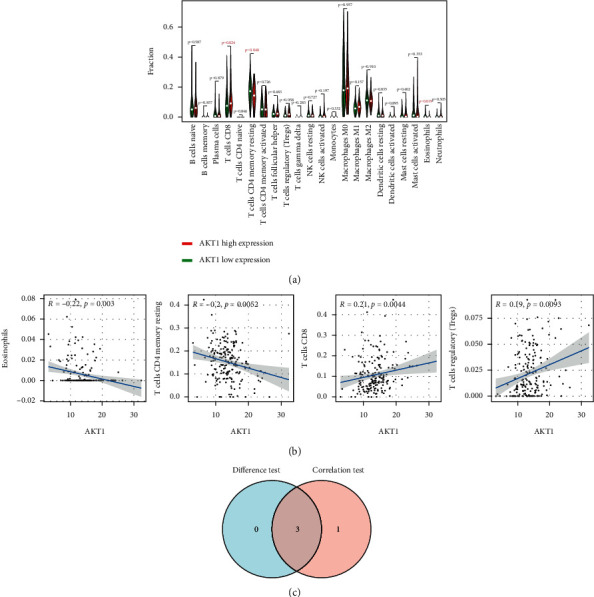
Correlation between TICs and AKT1 expression. (a) Violin figure shows the proportional differentiation of 22 immune cells relative to the expression level of AKT1 in CRC tissue, and the significance was tested by the Wilcoxon rank-sum method. (b) Scatter figure shows the correlation between TICs and AKT1 expression (*P* < 0.05). The blue line of each figure fitted the linear model between the proportional orientation of immune cells and AKT1 expression. Pearson's coefficient was used for the correlation test. (c) Venn figure shows three TICs related to AKT1 expression, which were determined by the difference test of violin figure and correlation test of scatter figure, respectively.

**Figure 9:**
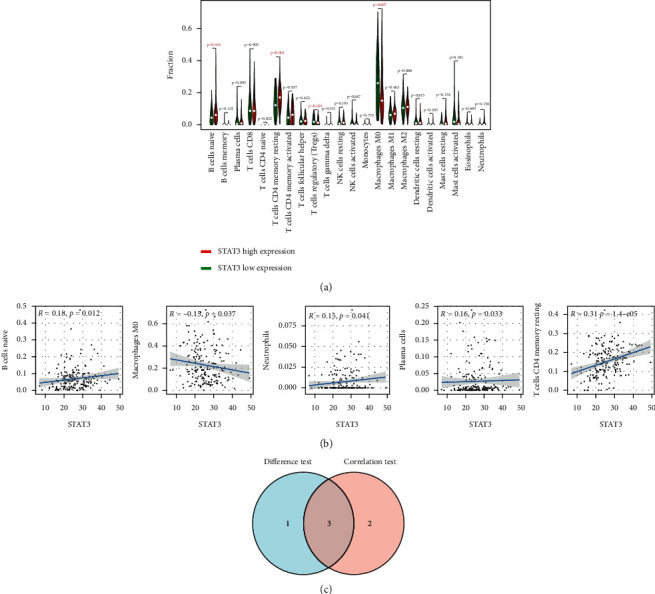
Correlation between TICs and STAT3 expression. (a) Violin figure shows the proportional differentiation of 22 immune cells relative to the expression level of STAT3 in CRC tissue, and the significance was tested by the Wilcoxon rank-sum method. (b) Scatter figure shows the correlation between TICs and STAT3 expression (*P* < 0.05). The blue line of each figure fits the linear model between the proportional orientation of immune cells and STAT3 expression. Pearson's coefficient was used for the correlation test. (c) Venn figure shows three TICs related to STAT3 expression, which were determined by the difference test of violin figure and correlation test of scatter figure, respectively.

**Figure 10:**
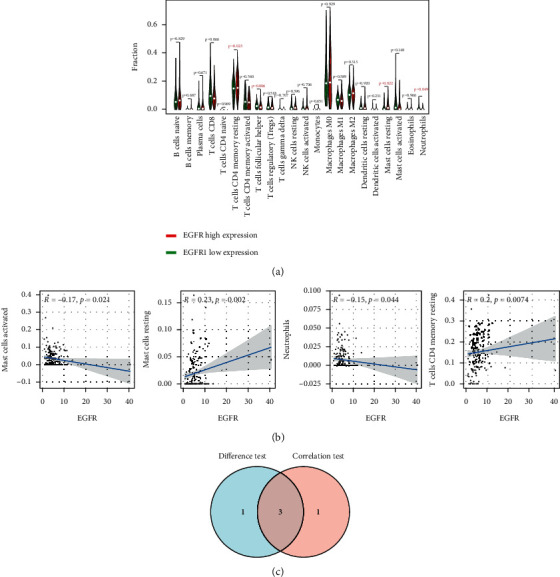
Correlation between TICs and EGFR expression. (a) Violin figure shows the proportional differentiation of 22 immune cells relative to the expression level of EGFR in CRC tissue, and the significance was tested by the Wilcoxon rank-sum method. (b) Scatter figure shows the correlation between TICs and EGFR expression (*P* < 0.05). The blue line of each figure fits the linear model between the proportional orientation of immune cells and EGFR expression. Pearson's coefficient was used for the correlation test. (c) Venn figure shows three TICs related to EGFR expression, which were determined by the difference test of violin figure and correlation test of scatter figure, respectively.

The CIBERSORT algorithm was used to analyze the proportion of tumor invasive immune subsets, and 22 immune cell maps of CRC samples were constructed (Figure 7). By Wilcoxon rank-sum test, violin curve (Figure 8(a)) showed that CD8+ T cells showed higher infiltration level in the high AKT1 group (*P* < 0.05), and T cells CD4 memory resting and eosinophils showed lower infiltration level in the high AKT1 group (*P* < 0.05). Using TIMER database to determine the relationship between screened genes and immune cell infiltration, we found that CD8+ T cells and T cells regulation were positively correlated with AKT1 expression (*P* < 0.05), and T cells CD4 memory resetting and eosinophils were negatively correlated with AKT1 expression (*P* < 0.05) (Figure 8(b)). B cells naive and T cells CD4 memory resting showed higher infiltration levels in the high STAT3 group (*P* < 0.05), while T cells regulatory and macrophages M0 showed lower infiltration levels in the high STAT3 group (*P* < 0.05) (Figure 9(a)). B cells naive, T cells CD4 memory resting, neutrophils, and plasma cells were positively correlated with STAT3 expression (*P* < 0.05), and macrophages M0 was negatively correlated with STAT3 expression (*P* < 0.05) (Figure 9(b)). T cells CD4 memory resting and Mast cells resting showed higher infiltration levels in the high EGFR group (*P* < 0.05), while T cells follicular helper and neutrophils showed lower infiltration levels in the high EGFR group (*P* < 0.05) (Figure 10(a)). T cells CD4 memory resting and Mast cells resting were positively correlated with EGFR expression (*P* < 0.05), while Mast cells activated and neutrophils were negatively correlated with EGFR expression (*P* < 0.05) (Figure 10(b)). According to the difference test and correlation test of violin diagram and scatter diagram, the Venn diagram shows three TICs related to the expression of AKT1, STAT3, and EGFR (Figures 8(c), 9(c), and 10(c)).

## 4. Discussion

CRC has become the second leading cause of cancer-related death in the world. The high degree of malignancy, rapid development, poor prognosis, and chemoradiotherapy resistance of CRC often lead to the failure of treatment. Previous studies have shown that curcumin is a promising candidate for the treatment of CRC [15–19]. However, the regulatory mechanism of curcumin in CRC treatment has not been systematically elucidated. Based on the “drug-target-pathway-disease” network [27], in this study, we explored the mechanism of action of curcumin in the treatment of CRC. We collected 30 potential targets of curcumin in the treatment of colorectal cancer from public databases. The GO function enrichment analysis showed 140 pathways; KEGG pathway enrichment analysis showed 61 signaling pathways, which were related to protein kinase activity regulation, negative regulation of apoptosis process, tumor signaling pathway, and PI3K-Akt signaling pathway. The results of molecular docking showed that curcumin can bind to AKT1, EGFR, and STAT3 more stably, and AKT1 has the strongest binding to curcumin.

By analyzing the PPI network and KEGG enrichment results, we predicted that AKT1, EGFR, and STAT3 were the core targets of curcumin in the treatment of CRC. Molecular docking analysis showed that curcumin had a good affinity for these three targets, and AKT1 had the highest binding degree. GO enrichment results showed that the therapeutic effect of curcumin was closely related to the regulation of protein kinase activity. Meanwhile, the KEGG enrichment results suggested that the PI3K-Akt signaling pathway played an important role, which indicated that this signaling pathway was the key link of curcumin in the treatment of CRC. AKT1, a member of the Akt family, is a serine/threonine protein kinase. Its abnormal expression plays an important role in the occurrence and development of a variety of malignant tumors [28–31]. PI3K-Akt is considered to be a key regulator of cell proliferation, angiogenesis, migration, and invasion that are related to the occurrence, development, and metastasis of tumors [32, 33].

Moreover, studies have shown that, under pathological conditions, epidermal growth factor receptor (EGFR) is the driving force of tumorigenesis, and it is considered as a biomarker of tumor drug resistance [34]. EGFR is a valuable therapeutic target for CRC, and EGFR inhibitors are effective drugs for the treatment of metastatic CRC [35], suggesting that curcumin may be used to treat metastatic CRC by inhibiting EGFR. STAT3 is involved in the microenvironment of tumorigenesis by secreting a large number of proinflammatory cytokines, which make the treatment of CRC difficult. STAT3 also plays an important role in the development of CRC [36], which is consistent with our study. The microenvironment is largely responsible for the response of CRC patients to therapy [37]. Our study shows that the key targets are closely related to TICs, which indicate that curcumin may regulate the tumor microenvironment and coordinate the balance between host and symbiont to achieve the homeostasis of the internal environment, maintain the body balance, and play the role of prevention and treatment of CRC.

In conclusion, in this study, we identified the key targets of curcumin in colorectal cancer inhibition through the combination of network pharmacology, molecular docking, and tumor immune microenvironment analysis. The mechanism of action of curcumin is binding to AKT1, STAT3, and EGFR by hydrogen bond, hydrophobic effect, and *π*-cation bond. This study provides a rational for further clinical research and new drug development using curcumin against CRC.

## Figures and Tables

**Table 1:** Molecular properties of curcumin.

Property	Value
Molecular weight	368.4 g/mol
Bioavailability score	0.55
XlogP3-AA	3.2
Hydrogen bond donor count	2
Hydrogen bond acceptor count	6
Rotatable bond count	8
PSA	93.1
Molar refractivity	102.80

**Table 2:** Topological parameters of the targets.

Targets	Degree	Subgraph	Betweenness	Closeness
AKT1	18	1576.9823	142.27713	0.78125
EGFR	15	1044.5238	123.55815	0.714286
STAT3	14	1046.328	76.63744	0.657895
EP300	12	1024.9966	26.278643	0.625
CDK4	10	789.8613	11.725108	0.595238
MMP9	10	728.5876	40.98225	0.609756
CHEK1	10	700.0684	27.078138	0.581395
GSK3B	9	602.72296	11.776262	0.568182
AURKA	9	560.9203	23.439177	0.555556
RPS6KB1	7	513.06287	1.7409091	0.555556
TOP2A	7	294.21695	9.762843	0.531915
EPHA2	7	255.23312	55.70498	0.555556
NFE2L2	5	301.88513	0.33333334	0.510204
TOP1	5	241.85707	0.5833333	0.510204
TLR9	5	241.24434	1.7222222	0.520833
HDAC4	5	220.83974	3.2777777	0.510204
RAF1	4	152.114	1.0666667	0.5
IKBKB	4	145.47293	1.7599567	0.5
EPHA3	4	43.854324	7.5857143	0.438597
EPHB2	4	37.902145	4.2099566	0.416667
BRAF	3	85.04361	0	0.480769
HDAC7	3	74.83233	0.5	0.471698
ADRBK1	3	63.870117	48	0.490196
MELK	3	56.340717	0	0.396825
EPHA7	3	14.385622	0	0.373134
MAOA	1	2.3126676	0	0.333333

**Table 3:** Molecular docking binding energies of core targets and compounds (kcal·mol^−1^).

Compound	AKT1	STAT3	EGFR
Curcumin	−9.9	−8.7	−8.5
Matrine	−7.8	−8.7	−7.6

## Data Availability

The raw data supporting the conclusion of this article will be made available by the authors without undue reservation.
